# Global analysis of protein synthesis in *Flavobacterium johnsoniae* reveals the use of Kozak-like sequences in diverse bacteria

**DOI:** 10.1093/nar/gkz855

**Published:** 2019-10-11

**Authors:** William D Baez, Bappaditya Roy, Zakkary A McNutt, Elan A Shatoff, Shicheng Chen, Ralf Bundschuh, Kurt Fredrick

**Affiliations:** 1 Department of Physics, The Ohio State University, Columbus, OH 43210, USA; 2 Center for RNA Biology, The Ohio State University, Columbus, OH 43210, USA; 3 Department of Microbiology, The Ohio State University, Columbus, OH 43210, USA; 4 Ohio State Biochemistry Program, The Ohio State University, Columbus, OH 43210, USA; 5 Department of Microbiology and Molecular Genetics, Michigan State University, East Lansing, MI 48824, USA; 6 Department of Chemistry and Biochemistry, Division of Hematology, Department of Internal Medicine, The Ohio State University, Columbus, OH 43210, USA

## Abstract

In all cells, initiation of translation is tuned by intrinsic features of the mRNA. Here, we analyze translation in *Flavobacterium johnsoniae*, a representative of the Bacteroidetes. Members of this phylum naturally lack Shine–Dalgarno (SD) sequences in their mRNA, and yet their ribosomes retain the conserved anti-SD sequence. Translation initiation is tuned by mRNA secondary structure and by the identities of several key nucleotides upstream of the start codon. Positive determinants include adenine at position –3, reminiscent of the Kozak sequence of *Eukarya*. Comparative analysis of *Escherichia coli* reveals use of the same Kozak-like sequence to enhance initiation, suggesting an ancient and widespread mechanism. Elimination of contacts between A-3 and the conserved β-hairpin of ribosomal protein uS7 fails to diminish the contribution of A-3 to initiation, suggesting an indirect mode of recognition. Also, we find that, in the Bacteroidetes, the trinucleotide AUG is underrepresented in the vicinity of the start codon, which presumably helps compensate for the absence of SD sequences in these organisms.

## INTRODUCTION

Initiation of translation requires the assembly of a ribosome complex with initiator tRNA bound to the P site and paired to the start codon of mRNA. Selection of the correct start codon among all other AUG (or similar) trinucleotides represents a critical challenge for the translation machinery. In *Bacteria* and *Archaea*, recognition of the start codon is often facilitated by the Shine–Dalgarno (SD) element, a purine-rich sequence (e.g. GGAGG) that lies ∼7–9 nt upstream from the start codon (1–3). The SD basepairs with a stretch of pyrimidines at the 3′ end of the 16S rRNA, termed the anti-SD (ASD) sequence, positioning the mRNA start codon in the 30S P site. Numerous genetic studies have shown that mutations that alter the SD or the spacing between the SD and start codon substantially reduce translation, demonstrating the importance of the SD for mRNAs that have them. However, not all prokaryotic mRNAs contain a SD sequence; for example, ∼10% of *Escherichia coli* mRNAs and ∼75% of *Caulobacter crescentus* mRNAs have no SD (4,5). Yet the translational machinery can still translate these SD-less mRNAs faithfully and efficiently, implying that other features of the translation initiation region (TIR) can facilitate start codon selection.

Genomic studies have revealed that SD sequences are completely absent in certain phyla of *Bacteria* (6). These lineages include the Bacteroidetes and a subset of Cyanobacteria. How the mechanism of translation initiation in these organisms differs from that of model organisms such as *E. coli* and *B. subtilis* remains unclear. It has been postulated that ribosomal protein S1, which interacts favorably with A/U-rich RNA, may functionally substitute for SD-ASD pairing in these lineages (6,7). Intriguingly, with rare exceptions (8,9), the 30S subunits of these SD-less organisms have retained the conserved ASD sequence at the 3′ end of 16S rRNA (6).

The Bacteroidetes represent a widespread and metabolically diverse group of microbes. Members of the Bacteroidetes are well known for their ability to digest and utilize various polysaccharides (10). In the mammalian gut, two phyla—Firmicutes and Bacteroidetes—account for the vast majority of bacteria present (11). Bacteroidetes within the gut break down many diverse glycans and hence alter the pool of nutrients that can be absorbed and utilized by the host. Indeed, human microbiome studies suggest important and complex contributions of the Bacteriodetes to nutrient acquisition, weight control, and metabolic disease (12–15). While these observations have spurred considerable interest in the Bacteroidetes, they remain an understudied group.

The Bacteroidetes exhibit unique aspects of not only translation but also transcription. The primary housekeeping sigma factor, termed σ^ABfr^ (after *Bacteroides fragilis*), has functionally diverged from the stereotypical σ^70^/σ^A^ of other bacteria (16). Sigma σ^ABfr^ lacks region 1.1 and contains numerous conserved substitutions in the DNA-recognition motifs of regions 2.3–2.4 and 4.2. A consensus promoter, with –33/–7 elements TTTG/TANNTTTG, has been deduced from various molecular studies (17–20). This promoter sequence differs dramatically from the –35/–10 elements recognized by E•σ^70^ holoenzyme (TTGACA / TATAAT), explaining why genes from *E. coli* are not expressed in the Bacteroidetes, and *vice versa* (21).

*Flavobacterium johnsoniae*, a member of the Bacteroidetes, is a common aerobic soil bacterium that can degrade chitin and other insoluble polymers (22). *Flavobacterium johnsoniae* exhibits gliding motility, and McBride and colleagues have developed *F. johnsoniae* as a model organism to elucidate the cellular machinery involved (22–27). In this work, we use ribosome profiling (RNA-seq and ribo-seq) to obtain a global snapshot of transcription and translation in *F. johnsoniae*. We find that translation initiation is tuned by mRNA secondary structure and by the identities of nucleotides upstream of the start codon. Positive determinants include adenine at position –3, reminiscent of the Kozak sequence of *Eukarya*. Comparative analysis of *E. coli* reveals use of the same Kozak-like sequence to tune initiation, suggesting widespread use of this mechanism across diverse lineages. The *F. johnsoniae* profiling data also allows refinement of the consensus promoter for the primary RNAP holoenzyme and predicts relative protein abundance across the proteome.

## MATERIALS AND METHODS

### Cell culture and library preparation

*Flavobacterium johnsoniae* strain UW101 was grown at 30°C to mid-logarithmic phase in rich CYE medium (28). To halt translation, cultures were rapidly chilled by mixing with crushed ice. Cells were collected by centrifugation and lysed via a freeze-thaw method (29). Ribosome-protected and total RNA fragments were isolated from the lysate, essentially as described previously (30). Briefly, to obtain the ribosome footprints, lysates were treated with 3 U/μl micrococcal nuclease (Roche) in the presence of 5 mM CaCl_2_ for 1.5 h at 25°C, and 70S monosomes were purified via sucrose gradient sedimentation. Proteins were extracted with phenol/CHCl_3_, RNA was precipitated with ethanol, and then RNA fragments in the 20–35 nt size range were gel purified. In parallel, cell lysates were directly extracted with TRIzol™ (Life Technologies), total RNA was subjected to limited alkaline hydrolysis, and fragments in the 30–50 nt range were gel purified. Corresponding cDNA libraries (ribo-seq and RNA-seq) were prepared as described (31).

### High-throughput sequencing, read processing and reproducibility

Sequencing was performed using a HiSeq2500 in the Ohio State Comprehensive Cancer Center Genomics Shared Resource in 50 basepair (bp) single-end mode. The three RNA-seq replicates initially contained 31 621 290, 30 635 429 and 29 764 896 single-end reads, and the three ribo-seq replicates initially contained 28 364 922, 17 598 576 and 27 203 601 single-end reads. 3′ adapter sequences were trimmed from the single-end reads using Skewer (version 0.2.2) (32) and its default settings, with the exception of the minimum allowed read length (‘−’) being set to 15. Sequences were then aligned to the *F. johnsoniae* genome (NCBI Reference Sequence: NC 009441.1) using the Bowtie 2 aligner (version 2.2.9) (33), using its default settings. Alignments were then converted to the BAM format using SAMtools (version 0.1.18) (34). Reads corresponding to ribosomal RNA (rRNA) and transfer RNA (tRNA) were removed from alignment files using the BEDtools (version 2.26.0) utility ‘intersect’ (35).

For the RNA-seq replicates, alignment efficiencies were 88.4% (65.2% multimapped), 88.1% (62.2% multimapped), and 88.5% (63.3% multimapped), of which 79.6%, 78.0% and 77.9% aligned to rRNA/tRNA genes. For ribo-seq replicates, alignment efficiencies were 81.6% (77.2% multimapped), 72.4% (68.7% multimapped) and 62.6% (60.1% multimapped), with 77.0%, 68.5% and 59.9% aligning to rRNA/tRNA genes. Analysis of gene expression (transcription and translation) entailed counting aligned RNA-seq fragments and ribo-seq footprints that overlapped coding sequence annotations using HTSeq (version 0.6.0) (36). Replicate-to-replicate comparisons of read counts per gene showed that the RNA-seq datasets were reproducible, with Spearman's correlation coefficients (*r*) of 0.982, 0.981 and 0.980 (Supplementary Figure S1). Analogous replicate-to-replicate comparisons showed that the ribo-seq datasets were also reproducible, with Spearman's correlation coefficients (*r*) of 0.958, 0.963 and 0.963 (Supplementary Figure S1).

### Calculation of average ribosome density (ARD)

For this study, we chose the highly expressed genes to act as a representative subset. To do this, we combined the data of the replicates and summed the per gene counts for RNA-seq fragments and for ribo-seq footprints. Then we rank-ordered (highest to lowest) the entire set of 5138 protein coding genes of *F. johnsoniae* by the number of RNA-seq fragments per gene length. From this list we arbitrarily selected the top third (1712 genes) as our representative set. Gene counts for both RNA-seq and ribo-seq were then normalized by the total number of counts per million from this representative set. Then, for each gene, we calculated the average ribosome density (ARD), the ratio of normalized ribo-seq footprint counts to normalized RNA-seq fragment counts, a parameter that largely reflects initiation rate (37–39). The genes were rank-ordered based on ARD and divided into octiles of 214 genes.

### Analysis of mRNA secondary structure

Regions around the annotated start codon of each analyzed gene were folded *in silico* and the average pairing probability for each nucleotide (nt) position was calculated. The ViennaRNA Package program “RNAfold" (40), with default settings, was used to calculate the pairing probabilities for each position. These pairing probabilities were then averaged over each set of genes.

### Comparative analysis of *E. coli* and *B. subtilis*

Published ribosome profiling (ribo-seq and RNA-seq) datasets from wild-type *E. coli* (GSE88725; (41)) and *B. subtilis* (GSE50870; (42)) cells grown under optimal conditions were similarly analyzed. We aligned and enumerated gene counts for *E. coli* and *B. subtilis* using the reference genome and annotations of NCBI NC 000913.3 and NCBI NC 000964.3, respectively. Analyzed gene sets contained 1440 genes (180 genes per octile) and 1392 genes (174 genes per octile), respectively.

### LacZ reporter gene experiments

DNA fragments, each containing a derivative of the P*_ant_* promoter (43) immediately upstream of the 5′ portion of *gene 32* of bacteriophage T4 (−21 to 13, where zero corresponds to the first nt of the start codon), without or with a basepair substitution, were generated by cassette mutagenesis (44), using oligonucleotides listed in Supplementary Table S1. Fragments were digested with EcoRI and BamHI, and cloned into the same sites of pRS552 (45). The resulting *gene 32-lacZ* fusions were transferred to λRS45 by homologous recombination (45) and integrated into the chromosome of *E. coli* strains BW25113 (46), CSH142 (47) and KLF3027 (48). A PCR-based method was used to confirm that each of the resulting strains contained a single prophage (49). β-Galactosidase activity was measured as described previously (50).

### GFP reporter gene experiments

The expression vector pSCH710, containing an IPTG-inducible σ^ABfr^-dependent promoter, was constructed in several steps. Overlap extension PCR (OE-PCR; (51)) was used to generate a DNA fragment containing the *ompA* promoter (P*_ompA_*) of *F. johnsoniae* (17,19) flanked by the operator sequences *lacO3* and *lacO1* of *E. coli*. This product was cloned into pGEM-T easy (Promega), generating pSCH685. Another chimeric DNA fragment containing the promoter for *rplC* (P*_rplC_*) from *F. hibernum* (18) upstream of the *lacI* gene of *E. coli* was generated by OE-PCR and cloned into pGEM-T easy, creating pSCH696. The Sac II–Sph I fragment of pSCH696 was then moved into the same sites of pSCH685, generating pSCH697. Finally, the Kpn I–Bam HI fragment of pSCH697, containing P*_rplC_*-*lacI* and *lacO3*-P*_ompA_*-*lacO1* in divergent orientations, was inserted into the same sites of pFj29 (17) to create pSCH710.

For TIR mutational analysis in *F. johnsoniae*, a ∼200 bp DNA fragment encompassing the TIR region of the EF-Tu gene (Fjoh_1936; chromosome region 2 238 784–2 239 021) was amplified from the *F. johnsoniae* chromosome and cloned into pDW01, a variant of pSCH710 in which the start codon of *gfp* is replaced by a Xho I site and six glycine codons. The resulting plasmid, pZM100, contains the 5′ portion of the EF-Tu gene translationally fused to the *gfp* reporter. Mutations in the TIR were introduced by Gibson Assembly (52), using the primers listed in Supplementary Table S1. Plasmids were moved into *F. johnsoniae* UW101 via tri-parental mating (28). For each strain, cells from overnight cultures were diluted 100-fold into CYE medium (3 ml) with erythromycin (100 μg/ml), and with or without IPTG (1 mM). Cultures were grown at 30°C for 6 h with shaking. Cells were pelleted, washed twice with PBS [137 mM NaCl, 2.7 mM KCl, 10 mM Na_2_HPO_4_, 1.8 mM K_2_HPO_4_ (pH 7.4)] and diluted in the same buffer to an OD_600_ of 0.4. For each cell suspension, fluorescence was measured with a Fluorolog-3 (HORIBA) spectrofluorometer, using an excitation wavelength of 481 nm and emission wavelength of 507 nm. Background, determined by measuring fluorescence of untransformed *F. johnsoniae* grown in parallel, was subtracted in each case. Reported values, Relative Fluorescence Units (RFU), represent fluorescence intensity per OD_600_.

## RESULTS

### Ribosome profiling

*F. johnsoniae* strain UW101 was grown at optimum temperature to mid-logarithmic phase in rich CYE medium. Cells were rapidly chilled to halt translation, sedimented, and lysed. Total RNA and ribosome-protected mRNA fragments were isolated in parallel from the lysate, and corresponding cDNA libraries were prepared and subjected to high-throughput sequencing. Replicate-to-replicate comparisons of read counts per gene showed that the RNA-seq and ribo-seq datasets were reproducible, with Spearman's correlation coefficients (*r*) in all cases greater than 0.98 and 0.95, respectively (Supplementary Figure S1). For this study, we chose the most highly transcribed genes (top third; 1712 genes) as a representative set for most analyses.

### RNA-seq data indicates that most transcripts are leadered

**Figure 1.**
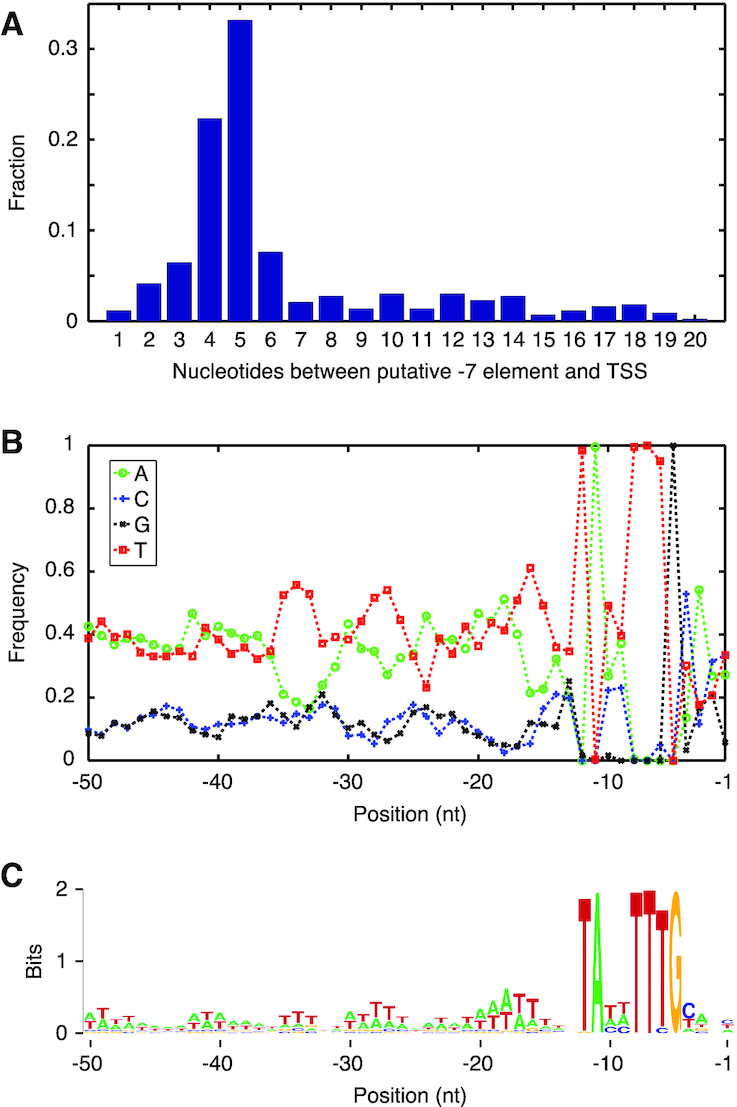
Analysis of promoters in *F. johnsoniae*. (**A**) A histogram showing the proportion of 434 candidate promoters with various distances (nt) between the TSS and potential -7 element. Those candidates with 4 or 5 nt spacing were deemed probable promoters. (**B**) 241 probable promoters were aligned with respect to the –7 element, and the average nucleotide frequencies (as indicated) across the region were calculated. (**C**) Sequence logo created from the data of panel B.

Leaderless mRNAs, characterized by an AUG start codon at (or nearly at) the 5′ end, are prevalent in certain *Bacteria* and *Archaea* (53–55). As the the absence of SD sequences could stem from the absence of leaders in *F. johnsoniae*, we used Rockhopper (version 2.03) (56) to predict transcriptional start sites (TSSs) from the RNA-seq data. Of the 1712 genes in our representative set, TSSs were predicted for 1532 (Supplementary Table S2). A sequence resembling the –7 promoter element characteristic of the Bacteroidetes was obvious just upstream from many of the predicted TSSs. We extracted the 50 nt immediately upstream of the predicted TSSs and used BioProspector (57) to search for promoters. 434 putative –7 elements were identified. For each, the distance to the TSS was calculated, yielding the histogram of Figure 1A. Many of the sequences have 4 or 5 nt between the –7 element and the start site, consistent with *bona fide* promoters (18,20). These 241 sequences (deemed probable promoters) were aligned with respect to the –7 element, and the nucleotide frequencies at each position were plotted (Figure 1B). Six nucleotides of the –7 element (T-12, A-11, T-8, T-7, T-6, G-5) are nearly invariant in this set of sequences (Figure 1B and C), consistent with previous work that defined the consensus TANNTTTG (17,18,20). As the occurrence of G at positions –10 and –9 is virtually zero, the –7 element consensus should be revised to TAHHTTTG (where H represents any nucleotide but G). A sequence motif corresponding to the previously described –33 element is much less evident. In fact, AT-rich motifs centered at –18 and –28 (consecutive turns of the DNA helix from the –7 element) are more pronounced than any motif near –33 (Figure 1C). Finally, for these genes with a probable promoter, mRNA leader length was calculated (Supplementary Table S2). Most (87%) of the predicted transcripts have a leader of more than 10 nt, and the overall average leader length is 33 nt. Thus, leaderless mRNA translation cannot explain the absence of SD elements in *F. johnsoniae*.

### Translation efficiency is tuned in part by mRNA sequence near the start codon

We calculated average ribosome density (ARD) for our representative set of 1712 genes in *F. johnsoniae* (Supplementary Table S2). A number of studies indicate that ARD (also termed ‘translation efficiency’ or ‘TE’) largely reflects the rate of initiation (37–39). Genes were rank-ordered by ARD and divided into eight groups, where octile 1 represents highest ARD and octile 8 represents lowest. Features of the TIR were then compared from octile to octile, in an effort to identify those that impact initiation.

**Figure 2.**
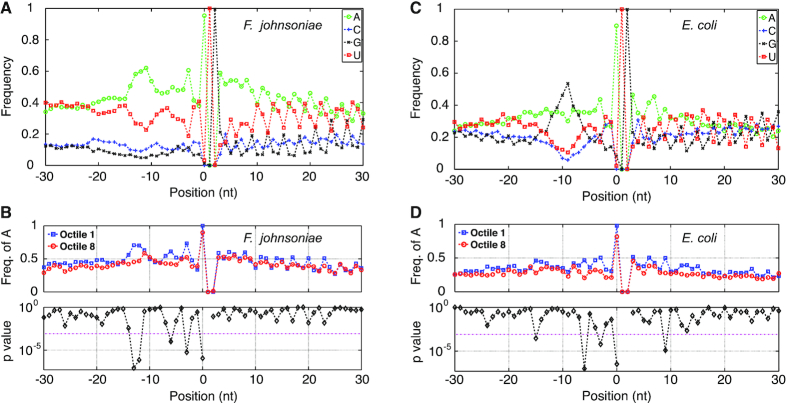
Adenines of the TIR act as positive determinants of translation in *F. johnsoniae* (**A, B**) and *E. coli* (**C**, **D**). Nucleotide frequencies (as indicated) for all analyzed genes are plotted across the TIR (A, C). Comparisons of the frequency of A in octile-1 versus octile-8 genes are shown below (B, D), with *P* values for each position calculated via a two-sample *t*-test. The dashed magenta line represents the Bonferroni-corrected significance threshold. Position 0 corresponds to the first nucleotide of the start codon.

**Figure 3.**
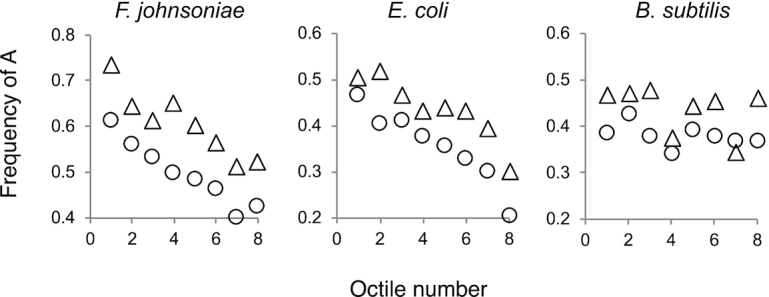
The frequency of A at positions –3 and –6 trends with ARD in *F. johnsoniae* and *E. coli*. Plots of A frequency versus octile in *F. johnsoniae, E. coli* and *B. subtilis* (as indicated). A-3, triangles; A-6, circles.

We looked at nucleotide frequencies at positions –30 to +30 of the TIR (where 0 corresponds to nt 1 of the start codon). A motif indicated by overrepresentation of adenines from –14 to –11 was evident (Figure 2A), as noted previously (5,58). Enrichment of As in this region correlated with ARD (Supplementary Figure S2A), with significantly higher frequencies of A at positions –12 and –13 in octile-1 genes compared to octile-8 genes (Figure 2B). This region of mRNA is predicted based on structural studies to lie in close proximity to ribosomal protein S1 when the start codon occupies the P site (59–62). Adenine frequencies at positions 0, –3 and –6 were also significantly higher in octile-1 versus octile-8 genes and generally correlated with ARD across all genes analyzed (Figure 2B, Supplementary Figure S2A, Figure 3). The frequency of U at positions 0 and –3 was significantly lower in octile-1 genes compared to octile-8 genes (Supplementary Figure S3A). Frequencies of C and G are generally lower across the TIR (Figure 2A), reflecting the low G+C content of the genome (34%) (22), and showed virtually no significant differences in octile-1 versus octile-eight genes (Supplementary Figure S3B and C). These data suggest that translation initiation is tuned in part by the first nucleotide of the start codon (position 0) and nucleotides upstream (positions –3, –6, –12, –13), with adenines in all cases acting as positive determinants.

For comparison, we similarly analyzed data from *E. coli* and *B. subtilis* to assess which nucleotides in the TIR influence initiation rate (Figure 2C and D, Supplementary Figures S4 and S5, Supplementary Tables S3 and S4). All-octile plots showed a prominent peak of guanines centered at -9 and –11 for *E. coli* and *B. subtilis*, respectively, indicative of the Shine–Dalgarno element (Figure 2C, Supplementary Figure S5). Yet comparisons of octile-1 versus octile-8 genes showed little or no significant difference in G enrichment in this region (Supplementary Figures S4 and S5). This is consistent with evidence that the strength of the SD is a poor predictor of initiation rate for endogenous genes in both *E. coli* and *B. subtilis* (39,63,64). For *E. coli*, adenines at positions 0, –3 and –6 were significantly more enriched in octile-1 versus octile-8 genes (Figure 2D), reminiscent of the *F. johnsoniae* case. Moreover, at these positions, A enrichment trends with ARD across all octiles (Figure 3, Supplementary Figure S2B). For *B. subtilis*, sequence differences between octile-1 and octile-8 genes were less evident, with the most compelling difference being higher enrichment of A at position 0 of octile-1 genes (Supplementary Figure S5). These data suggest that, in all three organisms, the first nucleotide of the start codon helps tune translation initiation. In *F. johnsoniae* and *E. coli*, nucleotide identity at positions –3 and –6 also matters, with adenines at both positions stimulating translation.

**Figure 4.**
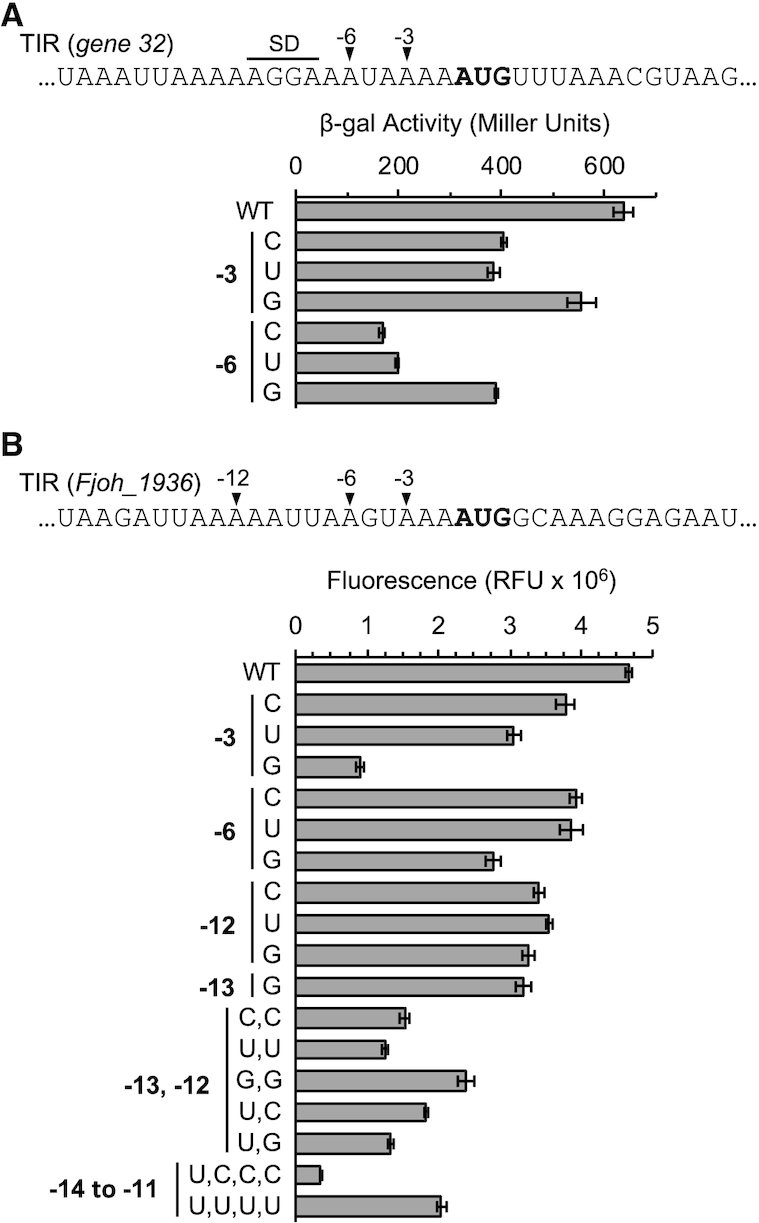
Functional analysis of upstream adenines in *E. coli* and *F. johnsoniae*. Effects of substitutions of A-3 and A-6 in the TIR of *gene 32* of phage T4 on translation were measured, using a single-copy *lacZ* reporter in *E. coli* (**A**). Effects of substitutions of certain adenines (single or multiple, as indicated) in the TIR of the EF-Tu gene (*Fjo_1936*) on translation were measured, using a plasmid-borne *gfp* reporter in *F. johnsoniae* (**B**). In the experiment of panel B, cells were grown in the presence of IPTG to induce transcription of the *gfp* gene. Bars represent the mean ± SEM of three or more independent experiments.

To test the functional importance of nucleotides at positions -3 and -6, we used reporter gene fusions. In *E. coli*, we fused the TIR of *gene 32* of phage T4 to *lacZ*, and integrated the fusion in single-copy into the chromosome. Analogous strains with the TIR mutated at position –3 or –6 were also made, and β-galactosidase (β-gal) activity of all strains was quantified (Figure 4A). The control (WT) TIR directed the highest level of β-gal production. Substitution of A-3 with either pyrimidine reduced the level of β-gal by ∼40%, whereas a smaller decrease (∼10%) was seen for A-3G. Mutation of A-6 was more deleterious, with decreases of ∼70%, 70% and 40% observed for A-6C, A-6U and A-6G variants, respectively.

In *F. johnsoniae*, we analyzed the TIR of gene Fjoh_1936, which encodes EF-Tu. Various derivatives of this TIR were fused to *gfp*, downstream of an IPTG-inducible promoter, on a plasmid shuttle vector. These plasmids were moved by conjugation into *F. johnsoniae*, and GFP fluorescence of the resulting strains was quantified (Figure 4B). Cells harboring the control (WT) construct and grown in the presence of IPTG gave the highest level of fluorescence. Substitution of A-3 with C, U, or G reduced GFP by 15, 30 and 70%, respectively. In this case, A-3G was particularly detrimental, suggesting an effect of the mutation beyond the loss of A, such as altered TIR folding. Mutation of A-6 also decreased GFP—by ∼15% for C/U and by 40% for G. Cells grown in the absence of IPTG exhibited much lower levels of fluorescence, yet effects of the TIR mutations were virtually identical (Supplementary Figure S6). These data show that both A-3 and A-6 can act as positive determinants of translation initiation in *E. coli* and in *F. johnsoniae*, as predicted from the ribosome profiling data.

The TIR of Fjoh_1936 has a run of adenines from –14 to –10, so we also targeted this region. Single substitutions of A-12 or A-13 reduced GFP production by ∼30%, and various double mutations reduced translation further (by 50–70%). Quadruple mutations replacing nt –14 to –11 with UCCC or UUUU reduced GFP by 90% and 60%, respectively. Why these mutations had differential effects remains unclear. But the fact that the latter mutation is no more detrimental than various double mutations suggests that the central adenines (A-12, A-13) are most critical, in line with the profiling data. Importantly, these data show that A-13 and A-12 can stimulate translation in *F. johnsoniae*.

### Roles of A-3 and A-6 in *E. coli* do not depend on the β-hairpin of ribosomal protein uS7

Cryo-EM structures of 30S preinitiation complexes (PICs) containing fMet-tRNA (PIC-2C, PIC-3, PIC-4, PIC-III) show a similar conformation of mRNA (65). In these complexes, mRNA nucleotides -3 to -6 contact ribosomal protein uS7, with the turn of its conserved β-hairpin packing against the A-3 base (Supplementary Figure S7). To test whether this interaction is responsible for discrimination of A-3, we moved each of the TIR-lacZ fusions into a strain of *E. coli* (KLF3027) in which the β-hairpin of uS7 is truncated (i.e. residues R77-Y84 are replaced with two glycines) (48) and into the isogenic control strain (CSH142). Essentially the same pattern of β-galactosidase activity was seen for the fusions, regardless of the genetic background (BW25113, CSH142 or KLF3027) (Supplementary Figure S8, Figure 4A). These data argue against direct recognition of A-3 by the ribosome.

### Translation efficiency is tuned in part by mRNA secondary structure

**Figure 5.**
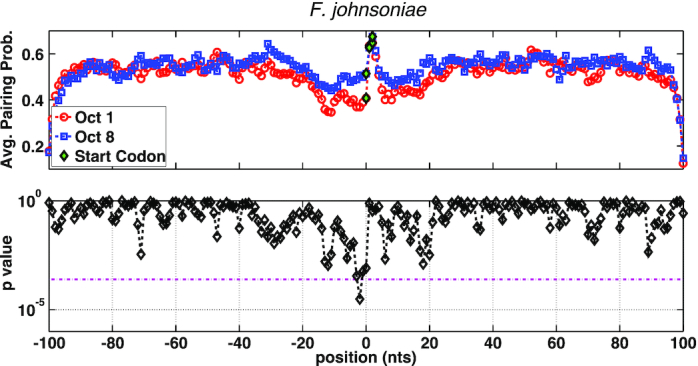
Translation in *F. johnsoniae* is tuned in part by mRNA secondary structure. Comparison of average pairing probability per TIR position for octile-1 (red) versus octile-8 (blue) genes. Corresponding *P* values (lower panel) were calculated via a two-sample *t*-test. The dashed magenta line represents the Bonferroni-corrected significance threshold.

A growing body of evidence indicates that TIR secondary structure plays a key role in initiation, with structured mRNA elements usually inhibiting the process (66–70). To assess the impact of structure, we folded each TIR *in silico* and then calculated the average probability of pairing for each nucleotide position. In *F. johnsoniae*, nucleotides in the vicinity of the start codon showed a reduced propensity for pairing, and this region of lowered structure (–20 to 20) surrounds the start codon in a fairly symmetrical fashion (Figure 5). Pairing probabilities for these nucleotides tended to be lower for octile-1 versus octile-8 genes, with *P* values near or beyond the Bonferroni-corrected significance threshold at several positions just upstream of the start codon. Similar trends were seen for *E. coli* and *B. subtilis* (Supplementary Figure S9). These data provide evidence that, in all three organisms, tuning of translation initiation is controlled in part by secondary structure within the TIR. One caveat here is that we considered only local folding (–100 to +100), based on *in silico* predictions, and hence our results may underestimate the role of secondary structure.

### Underrepresentation of AUG trinucleotides other than the start codon in the TIR

SD-ASD pairing is believed to facilitate start codon selection. Given this, it follows that an alternative mechanism(s) must be in place in organisms such as *F. johnsoniae* to enable start codon recognition. One simple way *F. johnsoniae* could reduce spurious initiation events would be to eliminate accessible AUG trinucleotides other than the start codon in each TIR. To investigate this idea, we quantified the number of AUG trinucleotides in the vicinity of the start codon relative to trinucleotides of the same base composition—GAU, GUA and AGU. For controls, we computed the analogous parameters at the gene midpoint.

In *F. johnsoniae*, the trinucleotide AUG is clearly underrepresented immediately upstream (−21 to −1) and downstream (4–24) of the start codon (Table 1). AUG/GAU ratios are ∼0.6 in the TIR windows, compared to ∼0.9 at midgene, suggesting that AUG trinucleotides in the vicinity of the start codon have been selected against in *F. johnsoniae*. We similarly analyzed *E. coli* and *B. subtilis* and found AUG trinucleotides to be more prevalent in the TIR (Table 1). In *E. coli*, the AUG/GAU ratios in the TIR (0.778, 0.757) are smaller than in the midgene windows (0.905, 0.901), suggesting some degree of underrepresentation. For *B. subtilis*, similar trends are observed, although AUG trinucleotides are more abundant in all windows. Qualitatively similar reductions in relative AUG frequency between TIR and midgene were found when normalizing to GUA and AGU. However, as these two trinucleotides contain the dinucleotides UA and AG, which are also present in stop codons, they are actually suppressed in the midgene windows, making these results harder to interpret.

**Table 1.** Relative frequency of the trinucleotide AUG near and far from the start codon

Ratio	TIR upstream^1^	TIR downstream^2^	Midgene upstream^3^	Midgene downstream^4^
*F. johnsoniae*				
AUG / GAU	0.616	0.605	0.905	0.956
AUG / GUA	0.569	0.736	1.633	1.673
AUG / AGU	0.568	0.587	1.590	1.642
*E. coli*				
AUG / GAU	0.778	0.757	0.905	0.901
AUG / GUA	1.135	1.082	1.792	1.842
AUG / AGU	1.010	0.948	1.919	1.899
*B. subtilis*				
AUG / GAU	0.851	0.898	0.941	0.957
AUG / GUA	1.740	1.562	2.322	2.337
AUG / AGU	1.470	1.310	2.235	2.321

^1^Positions -21 to -1.

^2^Positions 4 to 24.

^3^A 20 nt window 5′ of the midpoint of the gene.

^4^A 20 nt window 3′ of the midpoint of the gene.

We then extended this line of inquiry to include other Bacteroidetes, Proteobacteria and Firmicutes. We chose 10–12 representatives of each phylum (Supplementary Table S1) and conducted the same analysis as described above. For each phylum, we obtained *P* values using a one-sample t-test to assess any difference from one. To compare inter-phyla differences, we also performed a two-sample *t*-test for each combination of the three phyla. Furthermore, we adjusted *P* values with a Bonferroni correction for multiple test comparisons.

**Figure 6.**
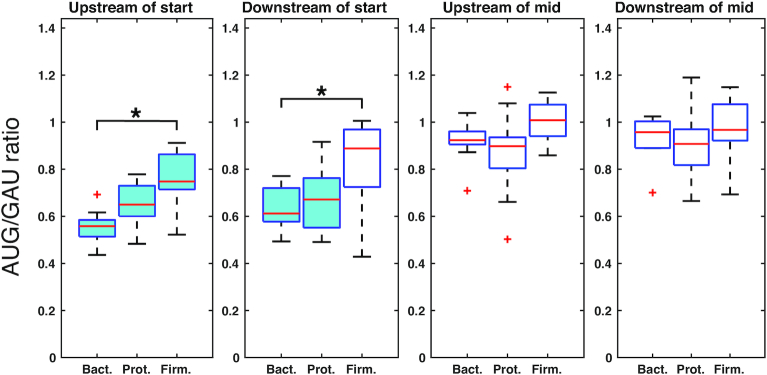
Underrepresentation of the trinucleotide AUG in the vicinity of the start codon in various bacteria. Shown is the frequency of AUG relative to GAU in 20 nt windows near or far from the start codon (as indicated) in the Bacteroidetes (Bact.), Proteobacteria (Prot.), and Firmicutes (Firm.), based on 10–12 representative genomes per phylum. Box-plot parameters: center line, median; box limits, upper and lower quartiles; whiskers, 1.5 times the interquartile range; points, outliers. Boxes shaded blue signify values statistically different from one and less than one (Bact., *P*_adj_ = 4.6 × 10^−8^ and 1.4 × 10^−7^; Prot., *P*_adj_ = 6 × 10^−6^ and 4.3 × 10^−4^; Firm., *P*_adj_ = 2.9 × 10^−3^). Brackets connecting two boxes indicate significant differences (*P*_adj_ < 0.05).

The AUG/GAU ratios were significantly different from 1 (and <1) (highlighted in blue) in the upstream window of the TIR for all three phyla (Figure 6). The ratio median was lowest for Bacteroidetes (0.56), intermediate for Proteobacteria (0.65), and largest for Firmicutes (0.75). In the downstream TIR window, the Bacteroidetes and Proteobacteria had AUG/GAU ratios significantly different from 1, and <1, which cannot be said of the Firmicutes. For both TIR windows, there was a significant difference in AUG/GAU between the Bacteroidetes and Firmicutes. In the midgene windows, AUG/GAU ratios for all phyla were statistically equivalent to 1 (Figure 6). Again, the data for the other two normalizing trinucleotides (Supplementary Figure S10) is harder to interpret due to their midgene suppression. Nevertheless, AUG frequency is significantly distinct from 1 (and <1) in the upstream TIR window for both normalizations and in the downstream TIR window for the AUG/AGU ratio in the Bacteroidetes. Collectively, these data show that the trinucleotide AUG tends to be underrepresented in the neighborhood of the start codon across all representatives of the Bacteroidetes. There is some indication of the same trend in the other phyla, particularly Proteobacteria. However, the degree of AUG underrepresentation in the TIR is largest in the case of the Bacteroidetes.

### Ribo-seq coverage predicts protein stoichiometries in *F. johnsoniae*

Weissman and colleagues have shown that relative protein levels in *E. coli* can be predicted with remarkable accuracy based on ribo-seq data (39). Because, to our knowledge, ours are the first ribosome profiling data for the Bacteroidetes, we calculated the relative rates of protein production in *F. johnsoniae*, as ribo-seq coverage per gene length, for all genes (Supplementary Table S2). These values estimate the stoichiometries of protein chains within complexes, such as those of the gliding-motility machinery, which includes a type IX secretion system (Supplementary Figure S11).

## DISCUSSION

How specific features of mRNA dictate the rate of translation initiation remains unclear. Studies of *E. coli* suggest that multiple parameters—including the SD sequence, start codon, spacing between the SD and start codon, and mRNA secondary structure—contribute, often in complex and intertwined ways (3,38,67,68,70–72). The complexity of the problem is exemplified by the fact that the free energy of SD–ASD pairing is a poor predictor of translation efficiency for endogenous mRNAs (39,63,64). For this work, we chose to analyze *F. johnsoniae*, which naturally lacks SD sequences, reasoning that other (non-SD) determinants might be unmasked.

We found that nucleotides at positions –3 and –6 help tune initiation in not only *F. johnsoniae* but also *E. coli*, with adenines acting as positive determinants at both positions. This is highly reminiscent of the Kozak sequence, which facilitates start codon recognition by the scanning 43S complex in *Eukarya*. Marilyn Kozak first characterized this sequence in vertebrates, deduced the consensus GCCRCCAUGG (where R represents A or G and the start codon is underscored), and showed that R-3 was functionally most critical (with A optimal) (73–76). More recent studies of diverse eukaryotes support the importance of R-3 and provide compelling evidence that the Kozak consensus stems from two distinct motifs—AAAAAAAUG and GCCGCCAUG (77,78). In lineages with AT-rich genomes, the former sequence predominates and hence defines the overall consensus, with A-3 being the most highly conserved nucleotide (77). Thus, the ability of A-3 to stimulate translation is widespread, suggesting a common mechanism across the Domains.

Cryo-EM structures of bacterial and eukaryal preinitiation complexes show direct contact between the conserved β-hairpin of uS7 and A-3 of mRNA (Supplementary Figure S7). This raised the hypothesis that an adenine at position –3 interacts most favorably with uS7 and thereby enhances initiation. However, ablation of this uS7-mRNA interaction fails to reduce the impact of A-3 (Supplementary Figure S8), arguing against direct discrimination by the small subunit. Hinnebusch and coworkers have also investigated the contribution of uS7 to initiation, using the yeast *S. cerevisiae* (79,80). They engineered many single-residue substitutions across the β-hairpin and evaluated the efficiency and accuracy of start codon usage in both poor (C-3) and optimal (A-3) Kozak contexts. The strongest phenotypes were conferred by substitutions in the ‘upper’ portion of the β-hairpin, well away from the loop residues that directly contact the mRNA (80). Further analysis showed that these mutations act by perturbing a key conformational transition (P_OUT_ to P_IN_) that occurs upon start codon recognition by the scanning 43S complex (79). Several substitutions in the loop increased the stringency of start codon selection (AUG versus UUG), although in a manner independent of the Kozak context (80). These data are in line with ours and suggest that Kozak / Kozak-like motifs are sensed in an indirect way.

How might the sequence upstream of the start codon influence initiation indirectly? As adenines have the lowest propensity for base pairing, the A-rich motifs could prevent formation of mRNA secondary structure that would otherwise hinder initiation. Another non-mutually-exclusive possibility is that the conformational dynamics of the RNA in single-stranded (ss) form influences initiation, and those sequences with favorable dynamics yield the consensus Kozak/Kozak-like motifs. It is known that the biophysical properties of ssRNA depend on sequence (81–87). Poly-A strands for example form right-handed A-type helices, which are stabilized by base-stacking interactions. By contrast, poly-U forms no such structures and instead behaves as a random coil. Poly-C forms helical structures resembling those of poly-A, although the poly-C structures have lower stability, presumably due to less favorable base stacking. While the conformational dynamics of heteropolymeric ssRNAs remain largely unexplored, it is tempting to speculate that the Kozak motifs AAAAAA and GCCGCC share key biophysical properties enabling them to functionally substitute for one another in eukaryal initiation. Intriguingly, the 3-nt periodicity of the latter matches that of the bacterial motif (A-3, A-6). Moreover, in PICs of *Eukarya* and *Bacteria*, the bases of nucleotides –1, –2 and –3 stack as a unit, separate from base-stacking interactions of mRNA nucleotides further upstream (65,88). It is reasonable to theorize that particular sequences would more readily adopt this conformation and hence have intrinsically higher affinity for this portion of the mRNA-binding channel of the small subunit.

We found that adenines further upstream of the start codon (−12, −13) also promote initiation in *F. johnsoniae*. Enrichment of adenines in this region is characteristic of the Bacteroidetes, and it has been speculated that ribosomal protein bS1 may bind this motif, functionally compensating for the absence of SD–ASD pairing in these organisms (5,6,58). Protein bS1 is a unique ribosomal protein, composed of multiple OB-folds (7), that reversibly associates with the 30S subunit. A recent cryo-EM study of the hibernating *E. coli* ribosome has revealed the position of bS1 (62), and superimposition of this structure onto that of the 30S PIC (which lacks bS1) (65) provides clues to how bS1 may contribute to initiation. Domain 1 of bS1 binds uS2, anchoring bS1 on the solvent-side of the subunit. The remainder of the protein extends around the platform toward the E site, with domain 4 of bS1 positioned to interact with mRNA nt –12 to –15. These observations are consistent with the hypothesis that the A-rich motif is recognized by bS1, but further experiments will be needed to test the idea directly.

Our work also points to a role for mRNA secondary structure in controlling initiation in *Bacteria*. We find that nucleotides in the vicinity of the start codon (–20 to +20) have generally lower propensity for pairing, an effect somewhat more pronounced in highly-translated genes. Although subtle, the evidence that TIR secondary structure tunes initiation rate is seen for all three bacteria examined. Given that adenines tend to reduce secondary structure, one open question is whether (and/or to what degree) differences in structure drive differences in sequence (e.g. nt –3, –6, –12, –13)—or *vice versa*. Further study will be needed to address this difficult question and elucidate precisely how these upstream adenines act.

In *Bacteria*, SD–ASD pairing is thought to play a critical role in positioning the start codon in the 30S site during initiation, raising the question of how the Bacteroidetes cope without SD elements. We hypothesized that these organisms may compensate in a simple way—by eliminating AUG trinucleotides other than the start codon in the TIR region. Consistent with this hypothesis, we show that the trinucleotide AUG is underrepresented in TIR, and the degree of underrepresentation follows the trend Bacteroidetes > Proteobacteria > Firmicutes. The opposite trend is seen for *dR_SD_* values (5,6), genome-wide measures of SD prevalence. These observations suggest the mRNA selection is simpler in the Bacteroidetes and may entail little more than rapid 30S binding to an unstructured TIR and lateral diffusion of the 30S PIC on the mRNA until the start codon is recognized. Indeed, in line with previous work (69), initiation may be largely governed by the folding and dynamics of each mRNA to idiosyncratically tune translation in these organisms.

## DATA AVAILABILITY

All of the sequencing data are available via the National Center for Biotechnology Information (NCBI) Sequencing Read Archive (SRA), under BioProject accession number PRJNA564991. Custom scripts used in the analysis of these data are available under GPL Version 3 from the corresponding author upon request.

## Supplementary Material

gkz855_Supplemental_Files
