# Supplementary material for: Global analysis of protein synthesis in Flavobacterium johnsoniae reveals the use of Kozak-like sequences in diverse bacteria
Source: Nucleic Acids Res. 2019 Oct 11;47(20):10477–88. doi: 10.1093/nar/gkz855 (PMC6847099; doi:10.1093/nar/gkz855)
Supplement: gkz855_Supplemental_Files [file gkz855_supplemental_files.zip › Baez-SUP.pdf]

SUPPLEMENTARY FIGURES (AND LEGENDS FOR TABLES S1-S4)

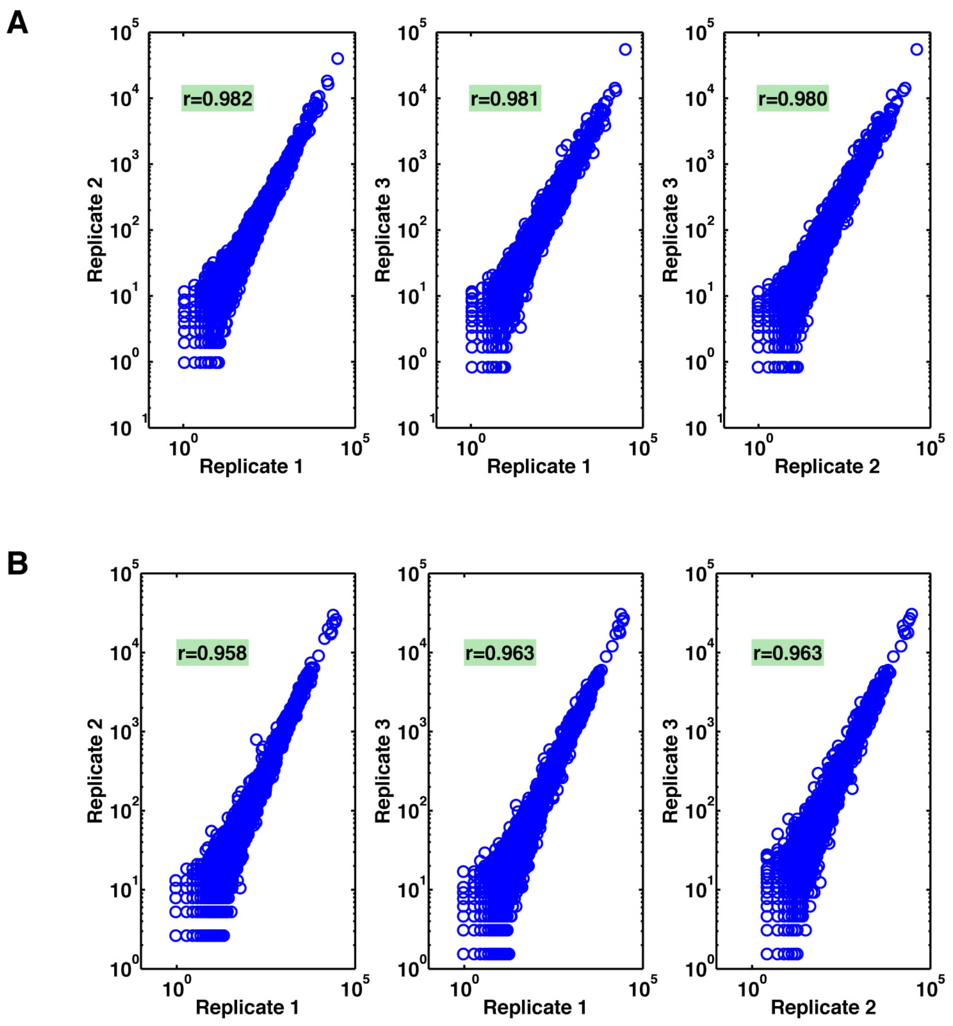

Figure S1. Reproducibility of RNA-seq and ribo-seq libraries. Pairwise comparisons of RNA-seq (A) and ribo-seq (B) per gene coverages for three biological replicates (as indicated) are shown. The Spearman's coefficient for each is listed, highlighted in green.

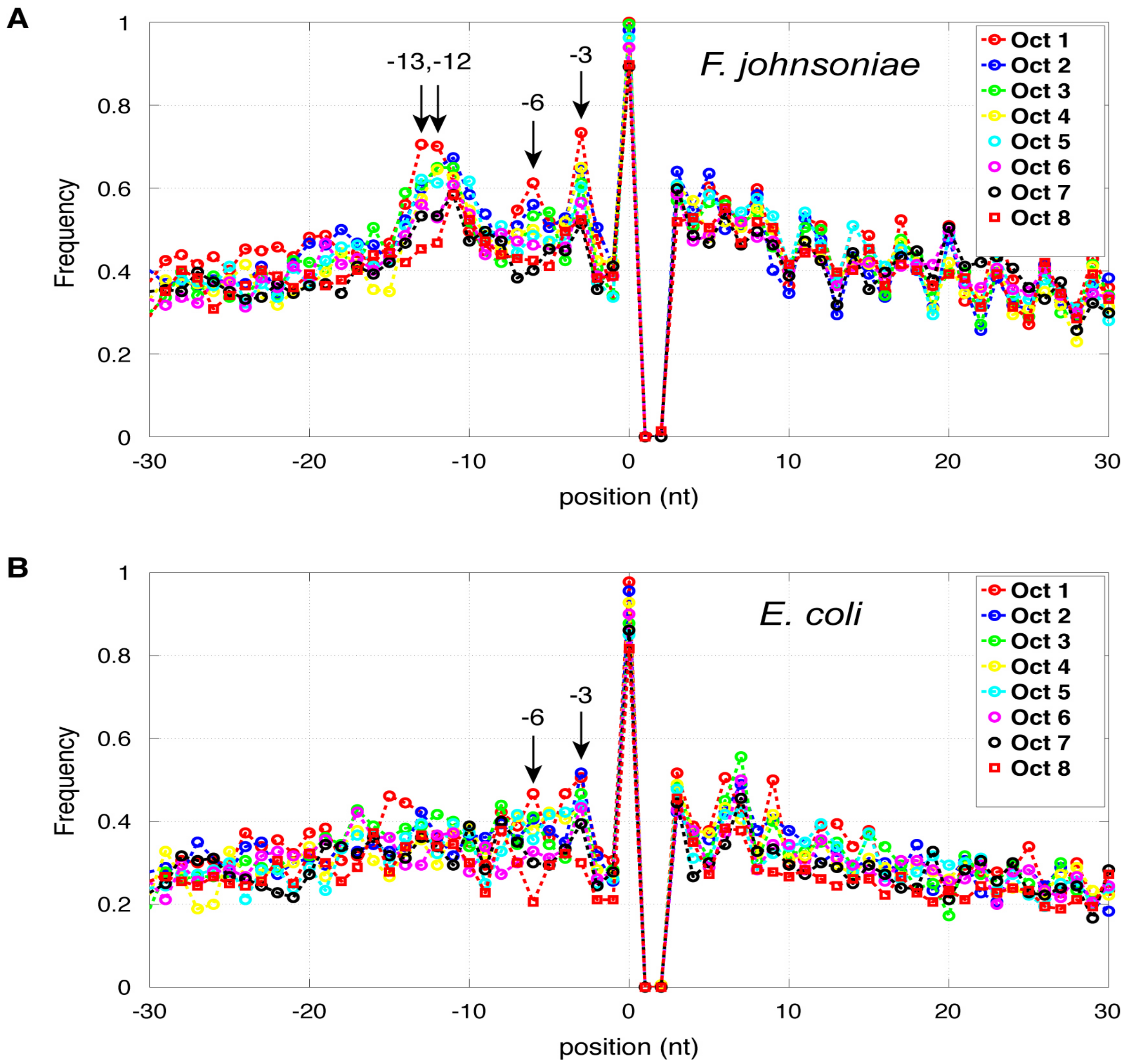

Figure S2. The frequency of A is plotted across the TIR for genes of octiles 1-8 (as indicated) of *F. johnsoniae* (A) and *E. coli* (B). Position 0 corresponds to the first nucleotide of the start codon.

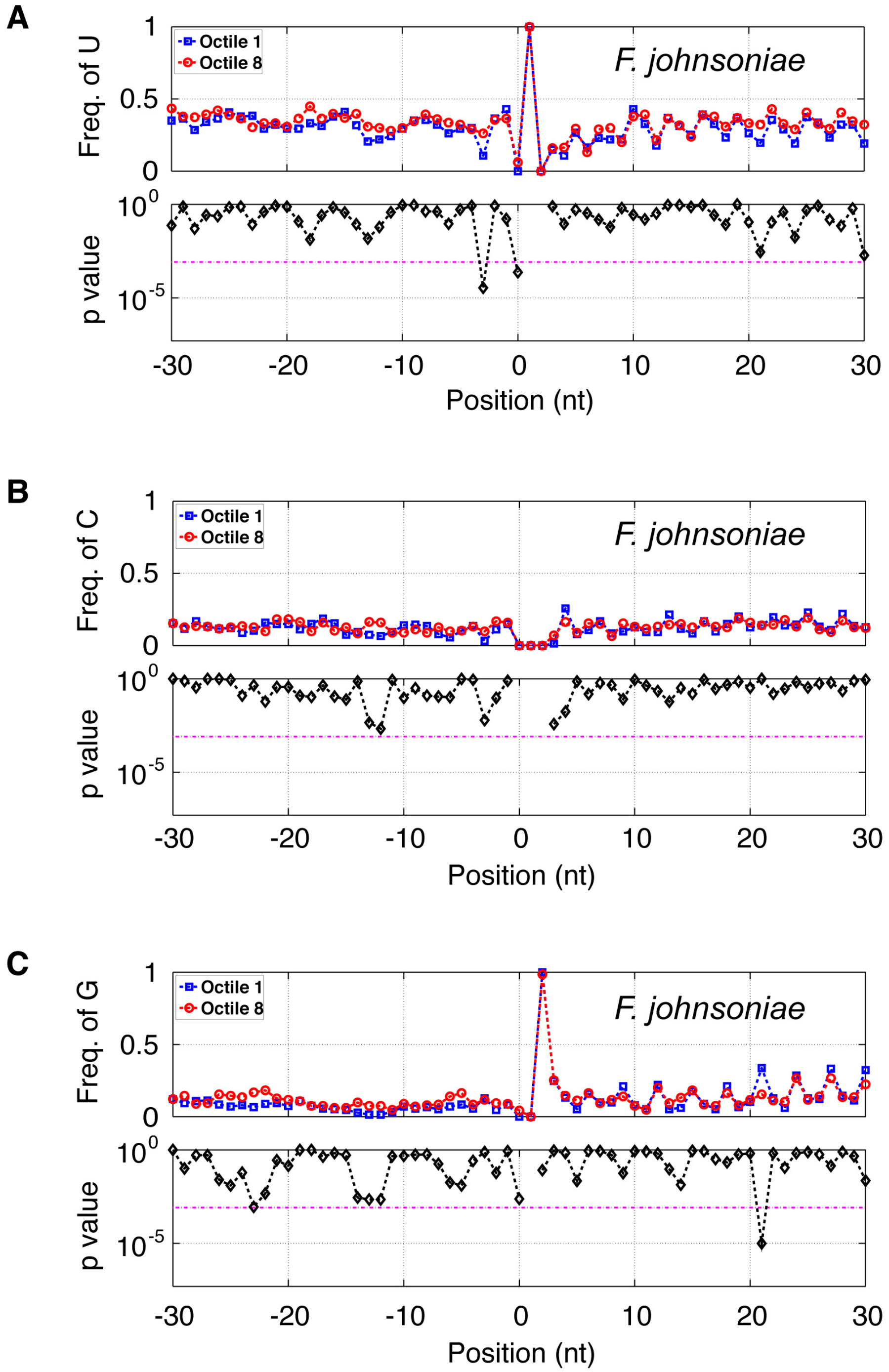

Figure S3. Comparisons of the frequency of U, C, and G across the TIR for genes of octile-1 versus octile-8 of *F. johnsoniae* (panels A, B, and C, respectively). The dashed magenta line represents the Bonferroni-corrected significance threshold. Position 0 corresponds to the first nucleotide of the start codon.

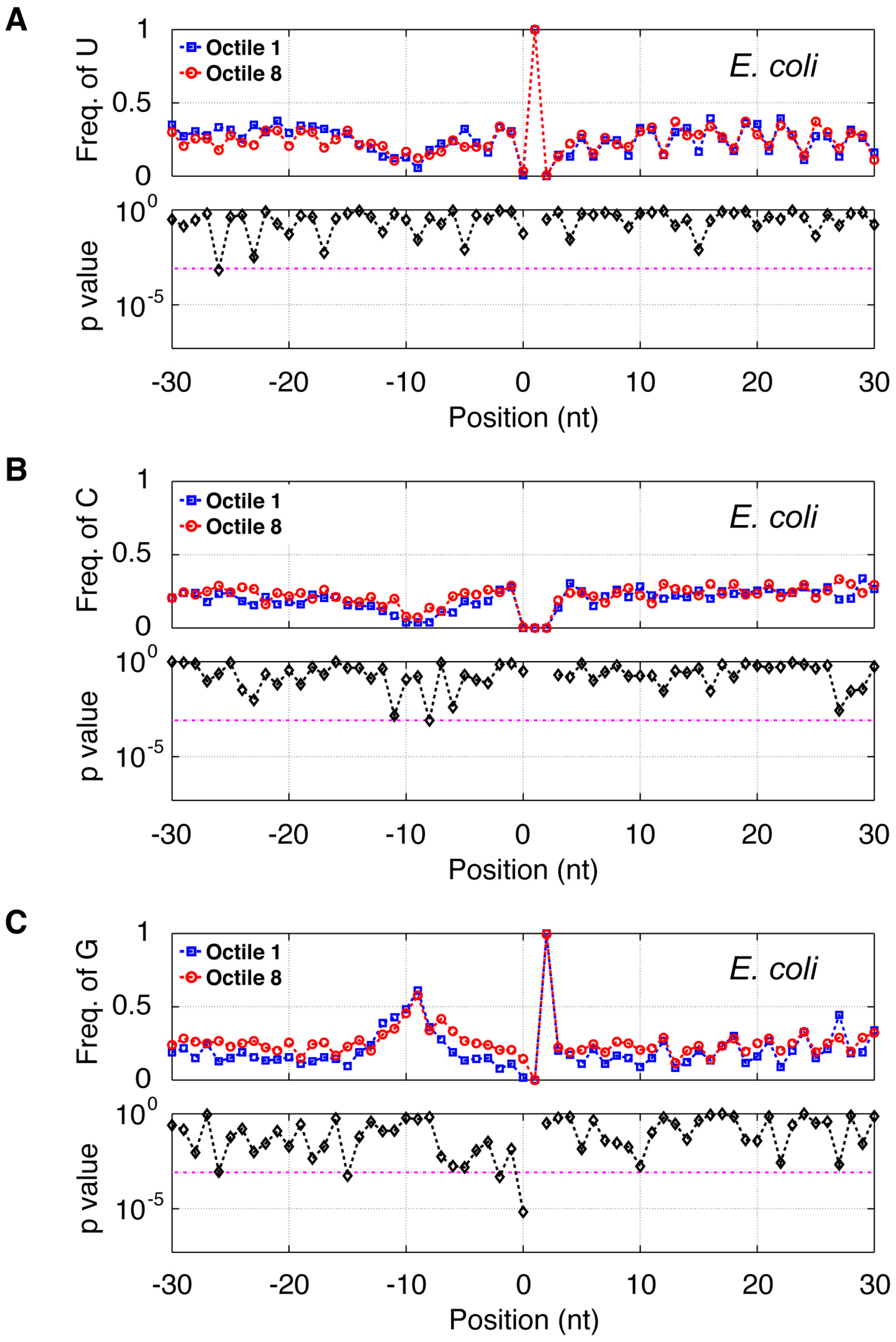

Figure S4. Comparisons of the frequency of U, C, and G across the TIR for genes of octile-1 versus octile-8 of *E. coli* (panels A, B, and C, respectively). The dashed magenta line represents the Bonferroni-corrected significance threshold. Position 0 corresponds to the first nucleotide of the start codon.

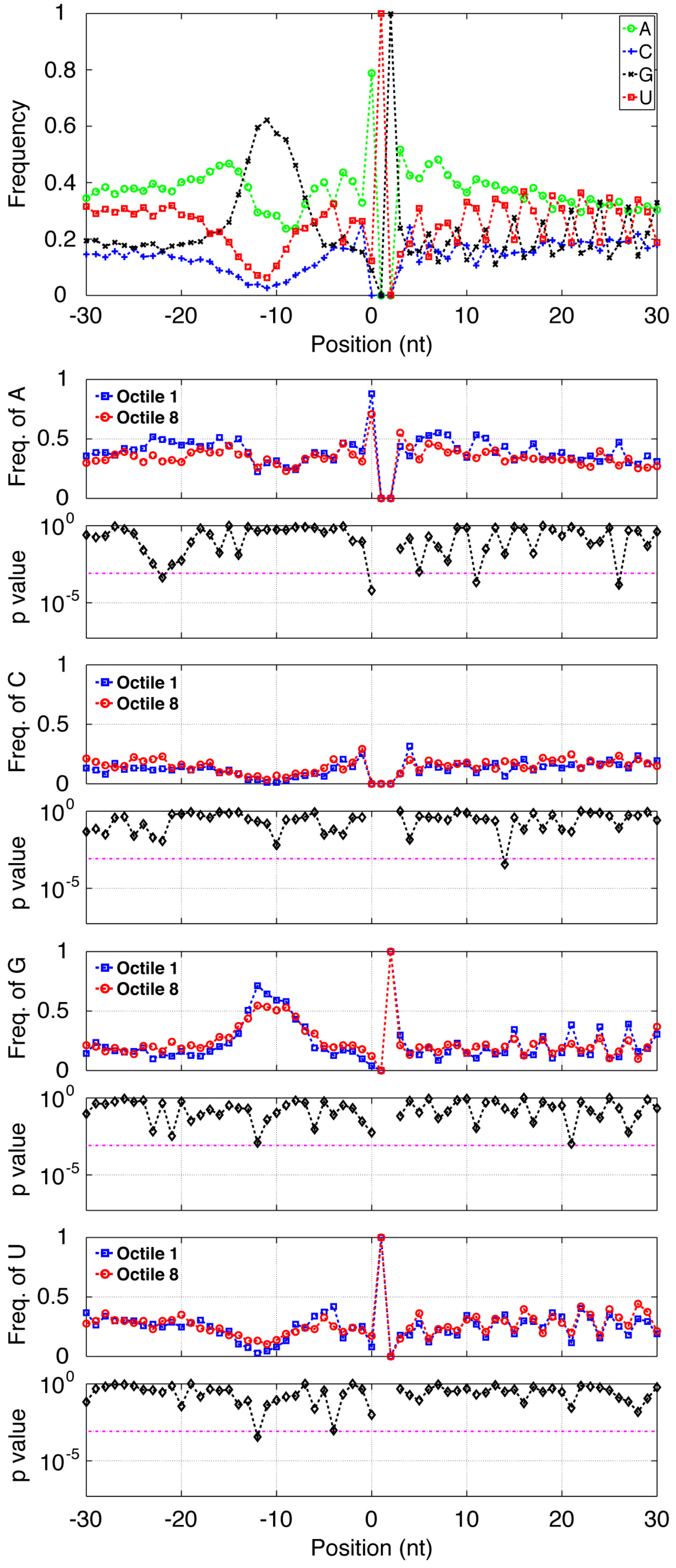

Figure S5. Analysis of mRNA sequence determinants that influence translation in *B. subtilis*. Nucleotide frequencies (as indicated) for all analyzed genes are plotted across the TIR (top panel). The frequency of A, U, C, and G across the TIR for octile-1 versus octile-8 genes are shown in lower panels (as indicated), with p values for each position calculated via a two-sample t-test. The dashed magenta line represents the Bonferroni-corrected significance threshold. Position 0 corresponds to the first nucleotide of the start codon.

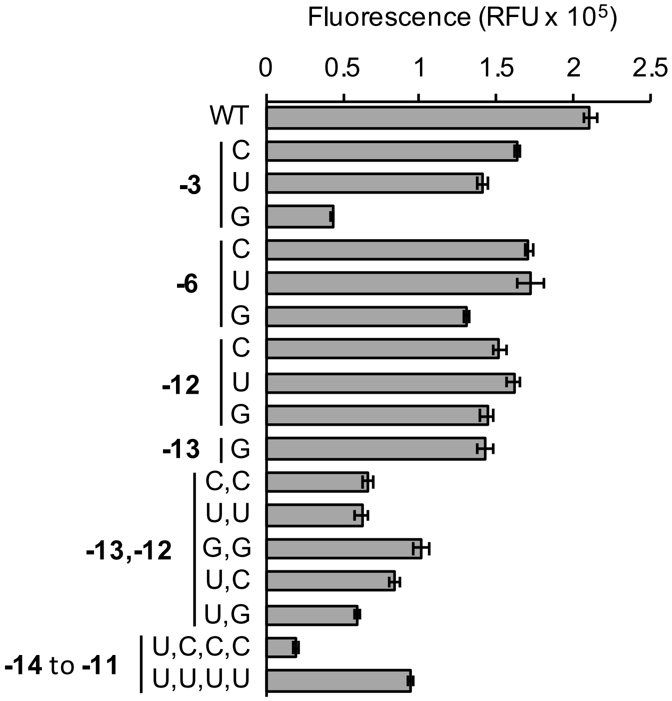

Figure S6. Effects of substitutions of certain adenines (single or multiple, as indicated) in the TIR of the EF-Tu gene (*Fjo_1936*) on translation were measured, using a plasmid-borne *gfp* reporter, in *F. johnsoniae* cells grown in the absence of IPTG. Data represent the mean ± SEM of three or more independent experiments.

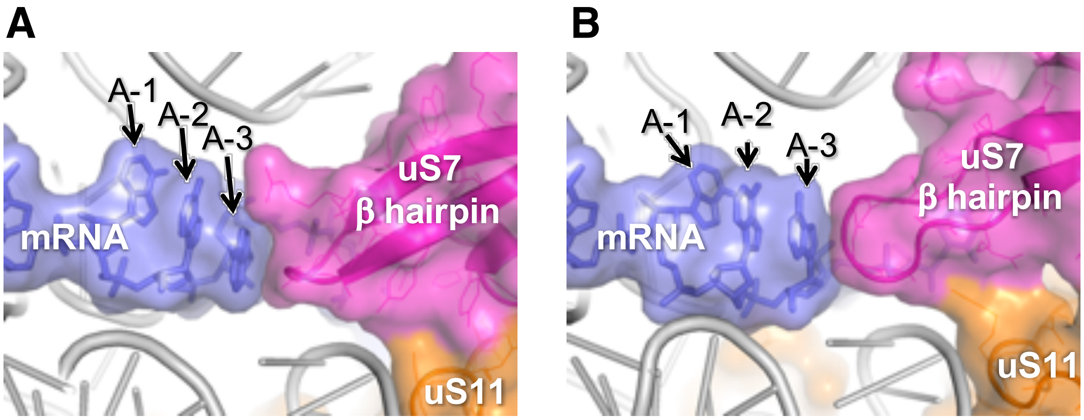

Figure S7. A ribosome-mRNA contact in the E site. The conserved β-hairpin of uS7 interacts with A-3 of mRNA in preinitiation complexes of *Bacteria* (A) and *Eukarya* (B). Images based on PDB files 5LMT and 6FYX.

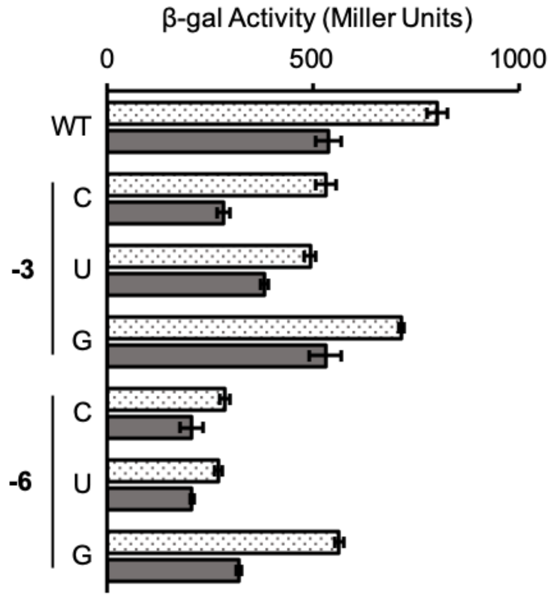

Figure S8. Removal of the uS7-mRNA contact has virtually no effect on mRNA selection. TIR(*gene 32*)-*lacZ* fusions without (WT) or with base substitutions (as indicated) were moved into CSH142 (control, stippled bars) or KLF3027 (S7ΔR77-Y84, gray bars), and β-galactosidase activities of the resulting strains were measured. Data represent the mean ± SEM of three or more independent experiments.

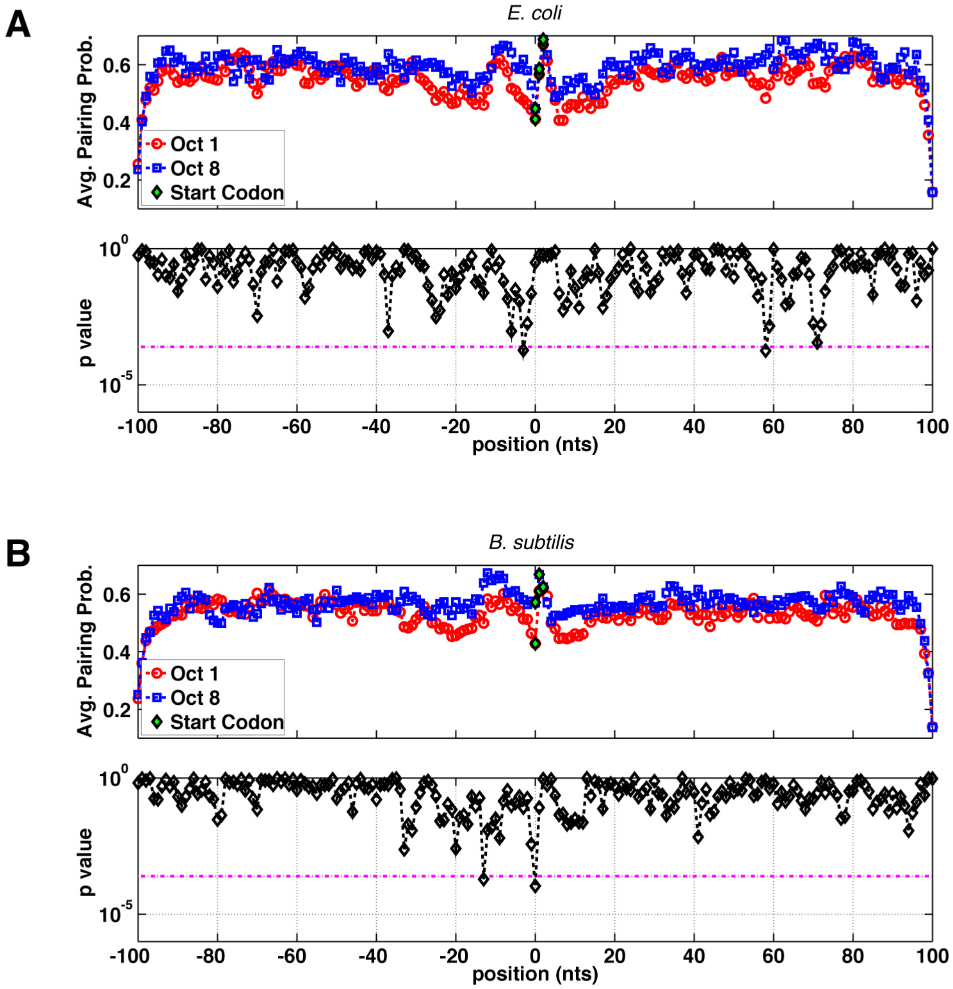

Figure S9. Secondary structure in the TIR helps tune translation in *E. coli* (A) and *B. subtilis* (B). Comparison of average pairing probability per position for octile-1 (red) versus octile-8 (blue) genes. Corresponding p values were calculated via a two-sample t-test. The dashed magenta line represents the Bonferroni-corrected significance threshold.

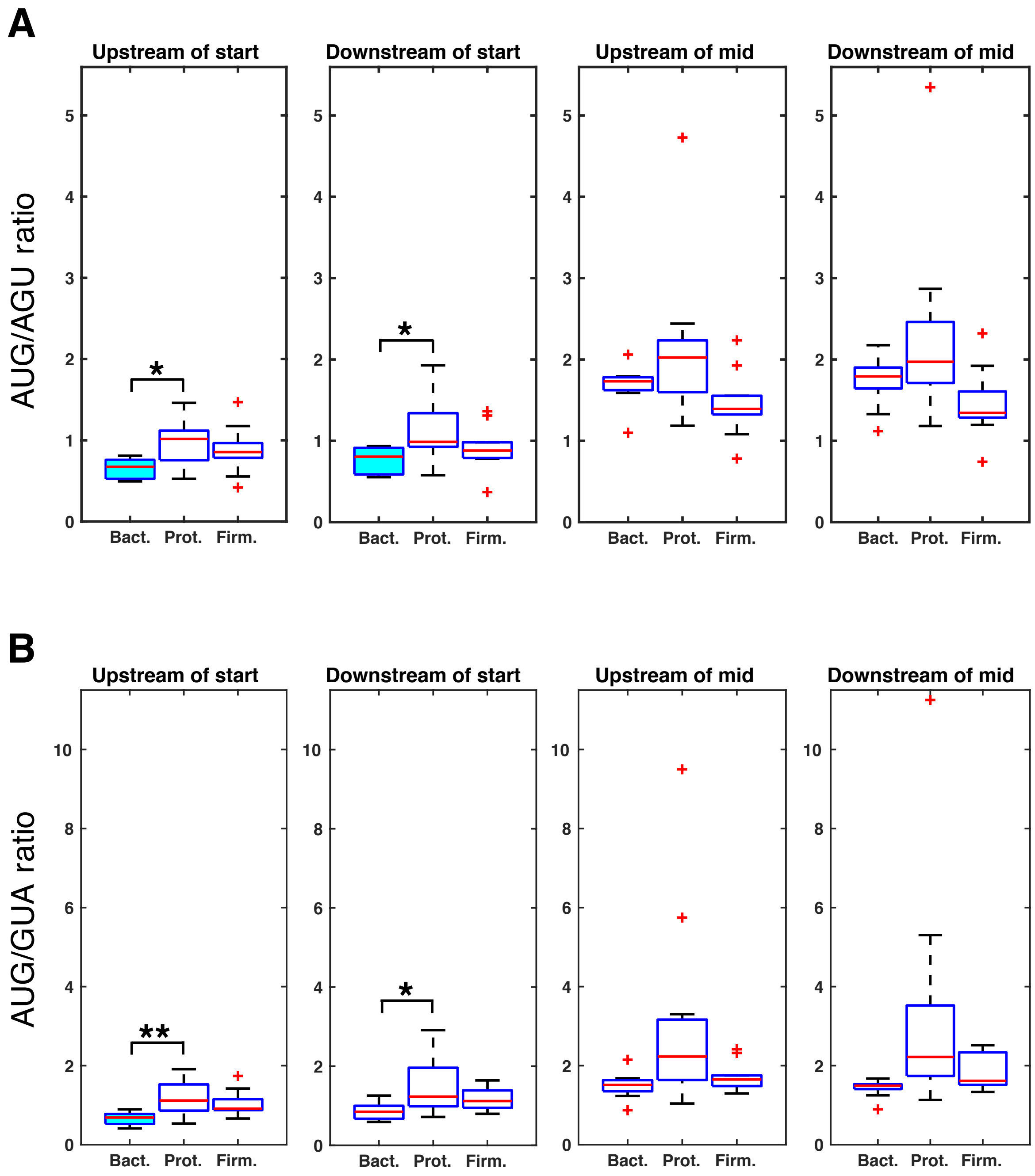

Figure S10. Evaluating the frequency of the trinucleotide AUG in the vicinity of the start codon in various bacteria. Shown are box plots depicting the relative frequency of AUG relative to AGU (A) or GUA (B) in 20 nt windows near or far from the start codon (as indicated) in the Bacteroidetes (Bact.), Proteobacteria (Prot.), and Firmicutes (Firm.), based on 10-12 representative genomes per phylum. Box-plot parameters: center line, median; box limits, upper and lower quartiles; whiskers, 1.5 times the interquartile range; points, outliers. Boxes shaded blue signify values statistically different from one and less than one ($p_{adj} < 0.05$). Brackets connecting two boxes indicate significant differences (*, $p_{adj} < 0.05$; **, $p_{adj} < 0.01$).

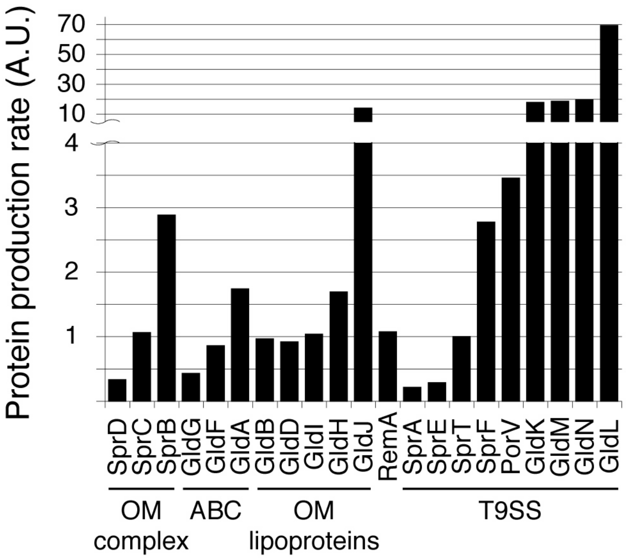

Figure S11. Predicting protein stoichiometries in *F. johnsoniae*. The relative rate of protein production from each gene of *F. johnsoniae* was estimated based on ribo-seq coverage. Inferred stoichiometries of components of the gliding motility machinery (as indicated) are shown. OM, outer membrane; ABC, ATP binding cassette transporter; T9SS, type IX secretion system. A.U., arbitrary units; normalized with respect to SprT.

LEGENDS FOR SUPPLEMENTARY TABLES

Table S1. Oligonucleotides (Tab 1) and representative genome sequences (Tab 2) used in this study.

Table S2. Summary of ribosome profiling data from *F. johnsoniae*. Tab 1 lists all protein-coding genes (columns A-G, self-explanatory) and reports total RNA-seq read count (column H), RNA-seq coverage per gene length (column I), total ribo-seq read count (column J), and ribo-seq coverage per gene length (column K). Values of column K predict the relative rates of protein production (in arbitrary units). Column L gives the standard errors for the values of column K, based on three biological replicates. Tab 2 lists the most highly-expressed genes (top third, based on RNA-seq coverage per length; columns A-G, self-explanatory) and reports normalized RNA-seq coverage (column H), normalized ribo-seq coverage (column I), average ribosome density (column J), Rockhopper-predicted transcription start sites (TSS, column K), candidate -7 elements (column L), distance from candidate -7 to TSS (column M), position of candidate -7G (column N), and leader lengths for those genes with promoters deemed probable (column O).

Table S3. Summary of ribosome profiling data from *E. coli*. Tab 1 lists all protein-coding genes (columns A-F, self-explanatory) and reports total RNA-seq read count (column G), RNA-seq coverage per gene length (column H), total ribo-seq read count (column I). Tab 2 lists the most highly-expressed genes (top third, based on RNA-seq coverage per length; columns A-F, self-explanatory) and reports normalized RNA-seq coverage (column G), normalized ribo-seq coverage (column H), and average ribosome density (column I).

Table S4. Summary of ribosome profiling data from *B. subtilis*. Tab 1 lists all protein-coding genes (columns A-F, self-explanatory) and reports RNA-seq coverage per gene length (column G), total RNA-seq read count (column H), and total ribo-seq read count (column I). Tab 2 lists the most highly-expressed genes (top third, based on RNA-seq coverage per length; columns A-F, self-explanatory) and reports normalized RNA-seq coverage (column G), normalized ribo-seq coverage (column H), and average ribosome density (column I).
